# Hernia recurrence as a problem of biology and collagen

**DOI:** 10.4103/0972-9941.27729

**Published:** 2006-09

**Authors:** Uwe Klinge, Marcel Binnebösel, Raphael Rosch, Peter Mertens

**Affiliations:** Department of Surgery, University Hospital of the RWTH Aachen, Aachen, Germany; * Department of Nephrology and Clinical Immunology, University Hospital of the RWTH Aachen, Aachen, Germany

**Keywords:** Biology, collagen, hernia, network, recurrence, wound healing

## Abstract

Usually an abdominal wall hernia is regarded as a mechanical problem with a local defect which has to be closed by technical means. Despite the introduction of several therapeutic improvements, recurrent hernias still appear in 10–15%. Therefore, reasons for a recurrence are discussed in a more fundamental way. It is assumed that a failure mainly depends on the quality of the repair. Correspondingly, in principle, the close causal relationship between the technical component and its failure during time is reflected by an s-shaped outcome curve. In contrast, the configuration of the outcome curve changes markedly if a breakdown is caused by numerous components. Then, the superposition of all incidence curves inevitably leads to a linear decline of the outcome curve without any s-shaped deformation. Regarding outcome curves after hernia repair, the cumulative incidences for recurrences of both incisional and inguinal hernia show a linear rise over years. Considering the configuration of outcome curves of patients with hernia disease, it may therefore be insufficient to explain a recurrence just by a failing technical repair. Rather, biological reasons should be suspected, such as a defective wound healing with impaired scarring process. Recent molecular-biological findings provide increasing evidence of underlying biochemical alterations in patients with recurrent hernia. Until predicting markers to identify patients with an impaired wound healing are available and considering the formation of insufficient scar as the underlying disease, the consequences for every surgical repair should be a supplementary reinforcement with nonabsorbable alloplastic nets as flat meshes with an extensive overlap.

Who or what is to blame for a recurrent hernia? The question whether the patient or the surgeon is responsible for a recurrence is of fundamental relevance not only in hernia repair but also in other therapeutic approaches, as in oncological surgery. Considering recurrence as a problem of biology and collagens, one should not neglect technical failures directly leading to a poor outcome.[1,2] However, regarding the persisting rates of operations for recurrent hernia of 10–15% worldwide despite all therapeutic improvements, a more fundamental pathology for hernia formation must be considered.[3–6]

## HERNIA RECURRENCE AS A TECHNICAL PROBLEM

Usually, an abdominal wall hernia is regarded as a mechanical problem with a local defect which has to be closed technically, either by sutures or, in modern times, with meshes.[7–9] In the long history of hernia repair, even the most experienced surgeon, irrespective of the utilized technique, has to face recurrences that have been created by him and correspondingly have to be regarded as his personal technical failure. Thus, why is it obviously impossible in hernia surgery to make mechanical repair with success rates being expected similar to those for engineering, without saying?

**Figure 1:**
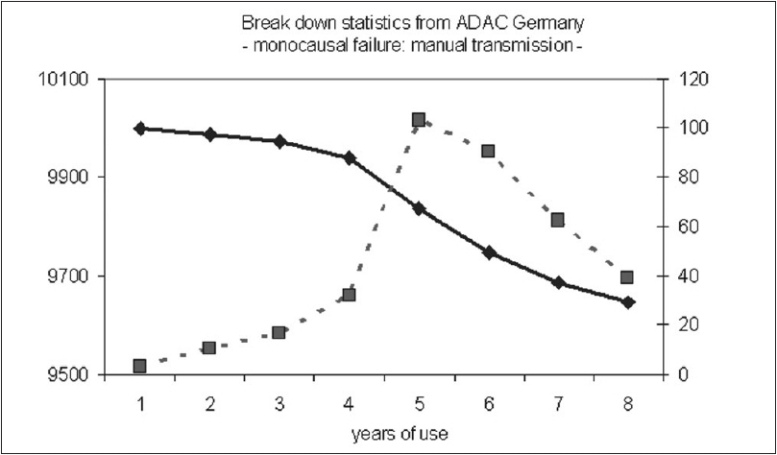
Statistical data from the general automobile club of Germany ADAC, depicting a mono-causal failure of manual transmission. The sshaped black curve is representing the age-related number of nondefective cars, whereas the cross lined curve illustrates the yearly failure of cars caused by a defective manual transmission with a peak incidence after 5 years (with courtesy of H. Schmaler, ADAC Munich, Germany).

**Figure 2:**
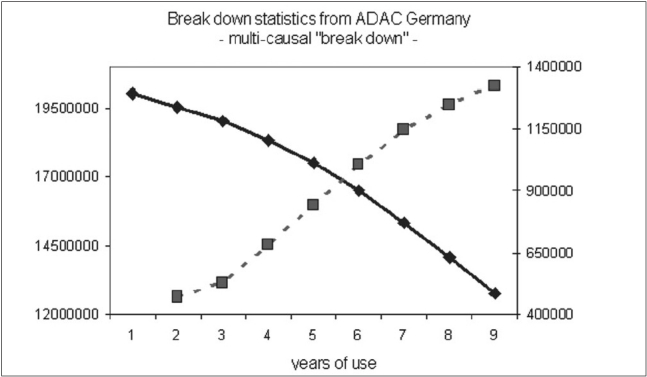
Statistical data from the general automobile club of Germany ADAC, representing a multi-causal failure. The black curve is representing the number of nondefective cars with regard to years of use. The cross-lined curve is illustrating the yearly failure of cars caused by various defects (with courtesy of H. Schmaler, ADAC Germany).

If a defective element in an apparatus is repaired by a technician, it is expected that it will work for some time, eventually breaking down again after a certain period of time. It is assumed that the occurrence of a failure just depends upon the quality of repair. For a large cohort of machines, the incidence of a breakdown of this element should represent a time-related maximum, when the repair stops working and correspondingly lead to an s-shaped ‘survival’-curve. In accordance, focusing on the breakdown of cars caused by one single component there results in an s-shaped survival curve with a maximum incidence after some years [Figure 1]. The close causal relationship between one technical component and its failure is reflected by s-shaped survival curve. Interestingly, the configuration of the survival curve changes markedly if a breakdown were to be caused by one of the numerous components of a car with incidences rising constantly over a period of time. It is the superposition of all survival curves that inevitably leads to a linear decline of the survival curve without any s-shaped deformation [Figure 2], disguising the causal impact of any one specified component.

What are the consequences for surgeons? Analogously, a close causal relationship between technique and failure may be expected only if an s-shaped survival curve is observed. If the recurrence is considered just as a technical failure, this should occur either soon or with a certain delay, but in any case the outcome curve should reveal an s-shaped configuration. However, this contradicts the actual proportions. In contrast, in incisional and inguinal hernia formation.[5,10,11] the cumulative incidences show a linear rise over years without any s-shaped deformation. This course is in contradiction to any significant direct causal relationship between technique and recurrence. Instead, an underlying multifactorial process has to be suggested. Furthermore, because most of the recurrences occur after 1 year within the linear rise of the cumulative incidences, a multifactorial process seems to be far more important than any accusable factor of the early postoperative course. Comparing the results of mesh repair with suture repair, it cannot even be concluded that mesh therapies in particular may only lead to a delayed manifestation of the recurrence in the long run instead of preventing them. For the latter case, it should be expected that the cumulative incidences should end in a final plateau. As a consequence, the consideration of recurrence as a result of a multifactorial and multi-causal process raises questions which indicate that complex system other than the operation procedure itself may serve as an explanation for this outcome.

## RECURRENCE AS A PROBLEM OF BIOLOGY AND COLLAGENS

**Figure 3:**
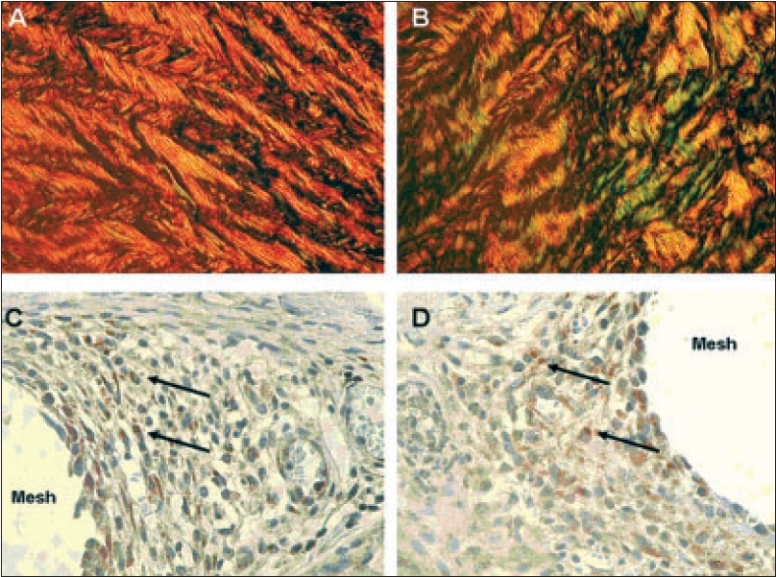
Cross polarization microscopical (CPM) and immunohistochemical features of human fascial tissue according to Junqueira.[12] CPM of Sirius red-stained section of normal fascia with a collagen type I/III ratio of 14 - (A); and specimen of recurrent incisional hernia fascia with a collagen type I/III ratio of 3.6 - (B). For the detection of MMP-2, we used rabbit polyclonal, 1:1000, from Biomol (Hamburg, Germany) as primary antibody; and goat anti-rabbit, 1:500, from Dako (Glostrup, Denmark) as secondary antibody. Positive cytoplasmatic expression of MMP-2 in granuloma adjacent to mesh filaments - (C, D) (positive stained cells marked with black arrows). (Magnification 400× in images IIIA - IIID).

Every technique used for hernia repair has to rely on formation of sufficient scar tissue. The scarring process is a form of defective healing replacing physiological tissues by fibrotic tissue abundant in fibroblasts and collagens. It results from a complex network with interactions of countless mediators of wound healing and an intensive cross-talk with cells, in particular with macrophages. Because of the long half-life of the collagens in comparison to all the various growth factors and cytokines, the collagens may reflect best the altered regulation of the scarring process, though changes in further components of the extracellular matrix have been observed, such as in the expression of the matrix metalloproteinase 2 (MMP-2) [Figure 3].[12–16] Therefore, it is not surprising that a decreased collagen type I/III ratio could be verified in adult patients with groin hernia (and in the scar of patients with recurrent hernia.[13,14] Whereas collagen type I is characteristic for mature scars or fascial tissue, the collagen type III represents the mechanically instable, less cross-linked collagen synthesized during the early days of wound healing. Correspondingly, in patients with recurrent hernias, there seems to be an impaired maturing of their scar tissue, which is not able to close the hernia gap or fix the mesh in place for long. As a consequence, a recurrence may develop either through a scar or at the border of a synthetic mesh through its scarry fixation.[17] Interestingly, an altered function could be detected even *in vitro* in macrophages’ free cultures of fibroblasts from patients with recurrent hernia, indicating an inherent and genetic and thereby probably systemic problem.[18] A comprehensive overview of the role of collagens in hernia disease is given elsewhere.[19]

**Figure 4:**
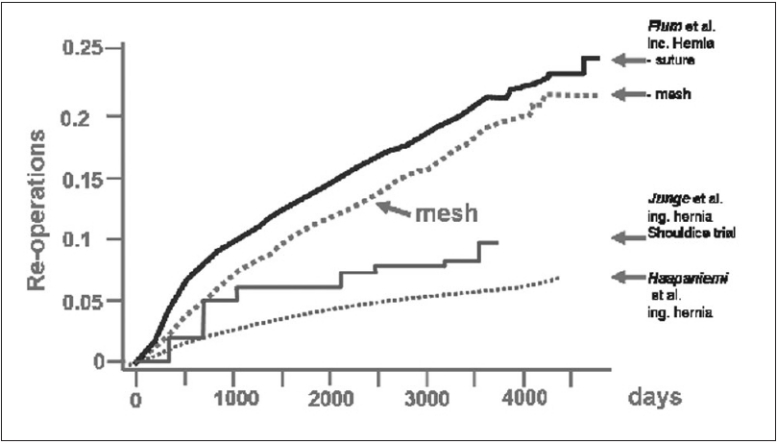
Cumulative incidences of recurrences after incisional and inguinal hernia repair[10,11]

The impaired quality of the scar of patients with recurrences not only explains the outcome curves [Figure 4] but also explains the fact that exogenous factors such as smoking could be identified as major risk factors.[20] Furthermore, it explains the high frequency of incisional hernia in patients with abdominal aortic aneurysm and their proven defect of the collagen metabolism.[21–23] It explains as well the frequent development of recurrences, if not the entire scar, was reinforced and that the best technique sometimes fails even in the hands of experts.

## TECHNIQUE OR BIOLOGY? IT IS BOTH!

Patients with hernia disease and in particular those with an incisional hernia are likely predisposed for recurrent hernia formation. Unfortunately, until now we do not have any predicting markers to identify those with an impaired wound healing and scar formation. The most significant factor still is a patient's history of hernia repair with markedly elevated re-recurrence rates.

Whereas recurrent and incisional hernias following suture repair are most likely caused by a defective biology, nevertheless the recurrence following mesh repair may be regarded as a technical fault, at least in theory. Despite the disappointing results in the study of Flum,[10] it should be achievable to delay a recurrence life-long, if a sufficient overlap is provided. In consideration of the tensile strength of current mesh materials, it is the extent of overlap which determines whether and when a recurrence may appear. In accordance, until now almost all recurrences manifest at the border of a dislocated, shrunken or undersized mesh, almost never through a mesh itself. Therefore, in principle it really should be possible to prevent recurrences by mesh repair, though until now this could not been proven by epidemiological data. However, there are a lot of personal series with excellent recurrence rates, underlining the efficacy of mesh repairs. A reason for this discrepancy of the results may be the neglect of the problem of overlap, either for anatomical reasons or for given limitations of some techniques. Focusing on a sufficient reinforcement of healthy tissue in all directions should improve recurrence rates.

## CONCLUSION

It is the consideration of an insufficient scar formation, at least in patients with recurrent hernia disease, that requires a supplementary reinforcement with nonabsorbable alloplastic nets as flat meshes with an extensive overlap; as a consequence, suture repair should be restricted to those cases in which a previous technical failure is likely, e.g., trocar hernia with missed fascia closure. Taking into consideration all patients with primary hernia, the experiences of the past decades with suture repairs indicate that we should expect 15–20% to develop a recurrent hernia. It will depend on the long-term biocompatibility of our mesh materials whether it is justified to apply a mesh repair to all of the patients or to restrict it for selected patients at risk. Future perspective may provide further possibilities to improve scar quality itself, e.g., by biological active meshes.
